# Octopamine receptor gene influences social grouping in the masked birch caterpillar

**DOI:** 10.1186/s13104-022-06102-3

**Published:** 2022-06-20

**Authors:** Chanchal Yadav, Jayne E. Yack, Myron L. Smith

**Affiliations:** Department of Biology, Carleton University, Ottawa, ON K1S 5B6 Canada

**Keywords:** Social, RNAi, Behavioral genetics, Grouping, Differential gene expression, Octopamine, *Drepana arcuata*, Insect

## Abstract

**Objective:**

Group-living plays a key role in the success of many insects, but the mechanisms underlying group formation and maintenance are poorly understood. Here we use the masked birch caterpillar, *Drepana arcuata,* to explore genetic influences on social grouping. These larvae predictably transition from living in social groups to living solitarily during the 3rd instar of development. Our previous study showed a notable shift in the *D. arcuata* transcriptome that correlates with the transition from grouping to solitary behavior. We noted that one differentially regulated gene, octopamine receptor gene (DaOAR), is a prominent ‘social’ gene in other insect species, prompting us to test the hypothesis that DaOAR influences grouping behavior in *D. arcuata*. This was done using RNA interference (RNAi) methods by feeding second instar larvae synthetic dsRNAs.

**Results:**

RT–qPCR analysis confirmed a significant reduction in DaOAR transcript abundance in dsRNA-fed larvae compared to controls. Behavioral trials showed that caterpillars with reduced transcript abundance of DaOAR remained solitary throughout the observation period compared to controls. These results provide evidence that regulation of the octopamine receptor gene influences social grouping in *D. arcuata*, and that specifically, a decrease in octopamine receptor expression triggers the larval transition from social to solitary.

**Supplementary Information:**

The online version contains supplementary material available at 10.1186/s13104-022-06102-3.

## Introduction

Group living is key to the survival of many insects, including many species of ecological and economical importance (e.g., [1–4]). However, the mechanisms associated with group formation and maintenance are poorly understood [4, 5]. To gain insights into such mechanisms we used the masked birch caterpillar (*Drepana arcuata*) as our study organism for two main reasons. First, during development these larvae predictably transition from living socially to solitarily, with the 3rd instar as the transition stage [6]. Second, a transcriptomic comparison between social and solitary phenotypes of *D. arcuata* showed differential expression of a number of genes associated with sociality in other organisms [7]. Among these, octopamine receptor (OAR) is of particular interest. Expression of OAR in gregarious and solitary *D. arcuata* showed a log fold change (log_2_FC) of 4.9, indicating a relative upregulation in early, gregarious instars [7]. Behavioral changes associated with octopamine receptors and the ligand, octopamine, are known to play a role in a variety of insect behaviors, including sociality [8–12]. In both the migratory locust, *Locusta migratoria*, and the desert locust, *Schistocerca gregaria,* for example, higher expression of octopamine receptor has been reported in gregarious compared to solitary phases [13, 14]. The differential expression patterns in *D. arcuata* together with associations with social behavior in other insects led us to test OAR function in *D. arcuata* larval grouping behavior.

In this study we test the hypothesis that a decrease in octopamine receptor expression triggers the larval transition from social to solitary. To test this, we used RNA interference (RNAi) to reduce octopamine receptor transcript abundance. There were two main goals: (1) To determine whether feeding synthetic dsRNA to *D. arcuata* larvae would elicit an RNAi response. This would be evident by a reduction in octopamine receptor gene transcript abundance based on RT–qPCR analysis and; (2) To test whether RNAi-induced reduction in octopamine receptor transcript altered the timing of the social to solitary transition in caterpillars.

## Main text

### Methods

#### Insect collection and rearing

Larvae were reared from eggs of female *D. arcuata* moths collected near Ottawa, Ontario, Canada (45.4215°N, 75.6972°W). Larvae were fed on paper birch leaves (*Betula papyrifera*) held in water-filled vials kept in glass jars at room temperature (21–23 °C) under 16 h light: 8 h dark lighting.

#### dsRNA design and synthesis

Isoforms of the octopamine receptor gene were previously found to be upregulated in socially grouping early instars of *D. arcuata* relative to solitary late instars [7]. After aligning the octopamine gene isoforms using Clustal Omega (https://www.ebi.ac.uk/Tools/msa/clustalo/), conserved regions were used for siRNA design using Dharmacon’s siDESIGN tool (https://horizondiscovery.com/en/products/tools/siDESIGN-Center). Double-stranded siRNAs were designed to match three different regions of the DaOAR transcript (Additional file 1: Table S1). For each region, sense and antisense 21-nucleotide ssRNAs were commercially synthesized (https://www.sigmaaldrich.com/life-science/custom-oligos/sirna-oligos.html) with a dTdT overhang at the 3^′^ end, individually dissolved in diethyl pyrocarbonate-treated (DEPC) water, and combined to allow formation of three dsRNAs, referred to as dsDaOAR-1, dsDaOAR-2, and dsDaOAR-3.

#### dsRNA feeding and behavioral trials

**Fig. 1:**
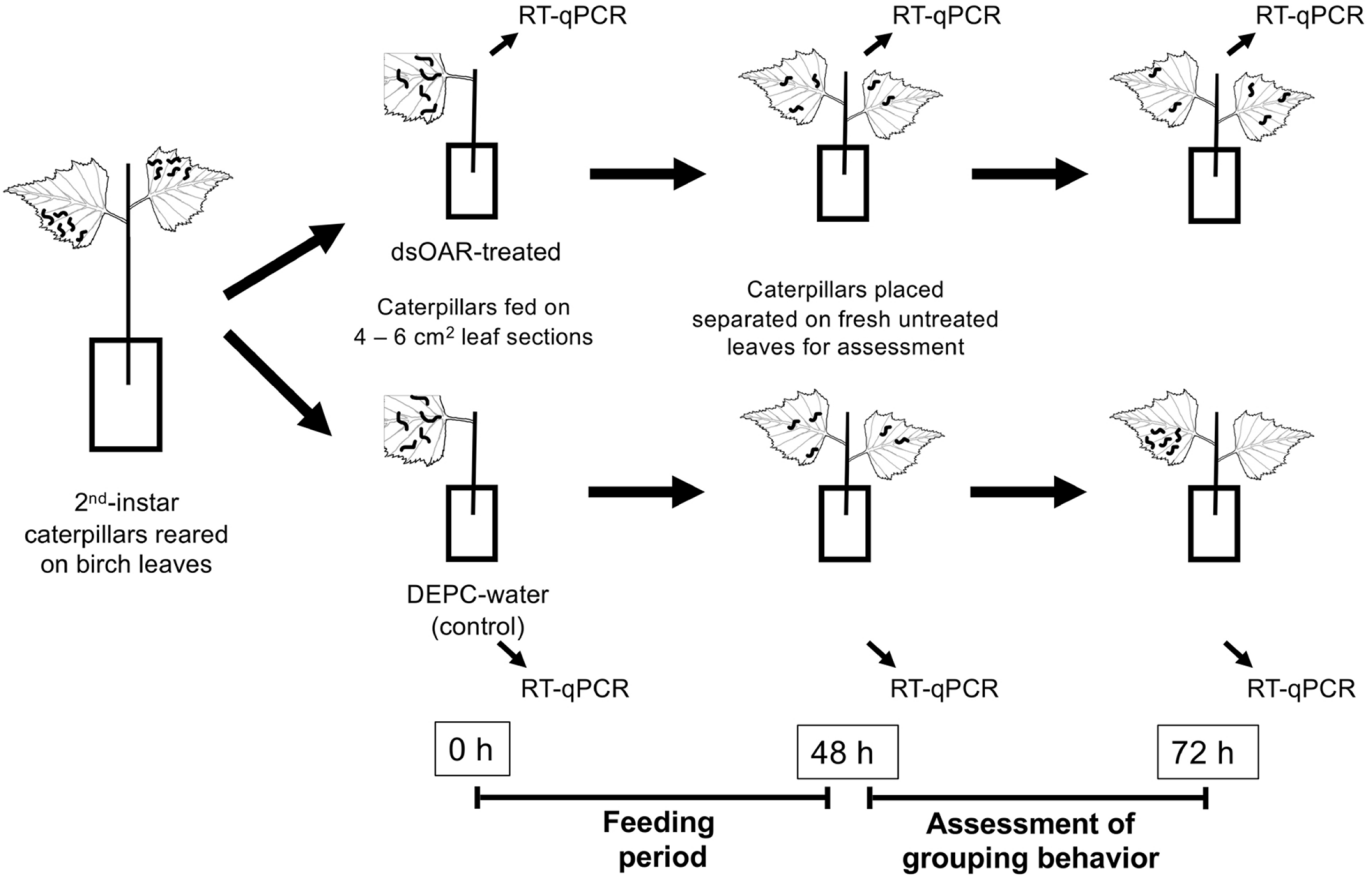
Overview of the methods used in this study. The effects of an octopamine receptor gene (DaOAR) on grouping behavior were tested by feeding second instar *D. arcuata* larvae synthetic dsRNAs

Second instar caterpillars, all within 1 day of molting into the 2nd instar, were used for the feeding and behavioural trials. There were two phases to the experiment—a feeding period on leaves coated in either dsRNA or DEPC-water control, and a behavioral assessment period (Fig. 1). Details on each trial including number of trials performed are included in Additional file 1: Table S3. At the beginning of each trial, a birch leaf (~ 4–6 cm^2^), was suspended by the petiole in a water-filled Eppendorf tube. Using a micropipette tip, 3 μg dsRNA (i.e. 2 μl volume) was applied evenly per 1 cm^2^ area on the upper surface of the leaf (dsOAR-treated), and an equal volume of DEPC water was applied to the upper surface of control leaves (Fig. 1). One hour after application of dsRNA or DEPC water (control), 2–3 larvae were flash frozen for qPCR and an additional 6–10 larvae were placed on the leaf (indicated by 0 h on Fig. 1) and allowed to feed for 48 h. At 48 h, 2–3 larvae were flash frozen for qPCR and the remaining 4–8 larvae were transferred, with each larva spatially separated, to a fresh sprig of 2–3 birch leaves (each leaf =  ~ 8 cm long × ~ 5 cm wide) held in a water-filled plastic vial to assess group forming behavior (Fig. 1). Grouping behavior was assessed for 24 h (i.e. between 48 and 72 h of the experiment (Fig. 1)), at the conclusion of which 2–3 larvae were flash frozen for qPCR. During the 24 h grouping assessment, larvae were monitored for the presence or absence of groups by monitoring the following parameters at the time points of 0, 1, 2, 3, 4, and 24 h: (i) the presence or absence of a group; (ii) number of groups established and; (iii) number of larvae in each group. Groups here refer to 2 or more individuals residing within an established silk shelter. The dsRNA treatment data and grouping data were each subjected to a Wilcoxon–Mann–Whitney test, as appropriate for small sample sizes, using R-package ‘stats’ in RStudio v1.3.1056 [15]. Leaf area consumed by caterpillars during feeding treatments was recorded to assess if there were any differences between control and dsOAR treatments.

#### Primer design and RT–qPCR

##### Primer design

RT–qPCR primers for a ‘housekeeping gene’, *DaRps7* (*D. arcuata* ribosomal protein 7), and octopamine receptor gene, *DaOar*, were designed using Primer3Plus v2.4.2 software [16]. Sequences of genes used to design primers were retrieved from the *D. arcuata* transcriptome assembly deposited at DDBJ/EMBL/GenBank under Accession Number GIKL00000000 [7] (see Additional file 1: Table S2 for RT–qPCR primer sequences). The PCR efficiency of each primer pair was comparable (~ 92%) as assessed using a 5-point standard curve.

##### RNA extraction and cDNA synthesis

To prepare samples for RNA extraction, a subset of two to three 2nd instars was flash frozen in liquid nitrogen at each of three time points during trials (Fig. 1): 0 h (beginning of feeding period), 48 h (end feeding period, just before behavioral observations), and 72 h (end of behavioral observations). Frozen caterpillars were stored at − 80 °C prior to RNA purification. Frozen caterpillars were ground in liquid nitrogen with a mortar and pestle followed by RNA purification using the Norgen Biotek Plant/Fungi RNA extraction kit (#31,350). DNase (Invitrogen TURBO DNase) was added during RNA extraction to eliminate residual DNA. Isolated RNA was quantified using a Nanodrop Spectrophotometer (Thermo Fisher Scientific, Waltham, MA, USA) and stored at − 80 °C in aliquots until cDNA synthesis. One μg of RNA was used for cDNA synthesis using M-MuLV reverse transcriptase and First Strand cDNA Synthesis Quick Protocol (New England Biolabs #M0253).

##### RT–qPCR

RT–qPCRs were performed using SYBR Fast Universal qPCR kit (Sigma-Aldrich). qPCR reactions were performed in duplicate with a total reaction volume of 20 μl [10 μl SYBR mix, 2 μl each of 10 μM forward and reverse primers (Additional file 1: Table S2), 4 μl milli-Q water and 2 μl of cDNA]. qPCR was carried out using Bio-Rad’s CFX Connect system (Bio-Rad Laboratories, Hercules, California, USA) with the following thermal cycling conditions: initial denaturation at 95 °C for 3 min followed by 40 cycles of 95 °C for 10 s, 60 °C for 15 s and 72 °C for 20 s. No-template controls and no-reverse-transcriptase controls were used during each qPCR run to confirm the absence of primer-dimer formation and DNA contamination, respectively. RT–qPCR values across trials were normalized to the reference gene (*DaRps7*) and the double delta Ct method was used for relative quantification of octopamine transcript abundance in dsRNA-treated vs control caterpillars [17].

### Results

#### RNAi-associated decrease in octopamine receptor transcript

**Fig. 2:**
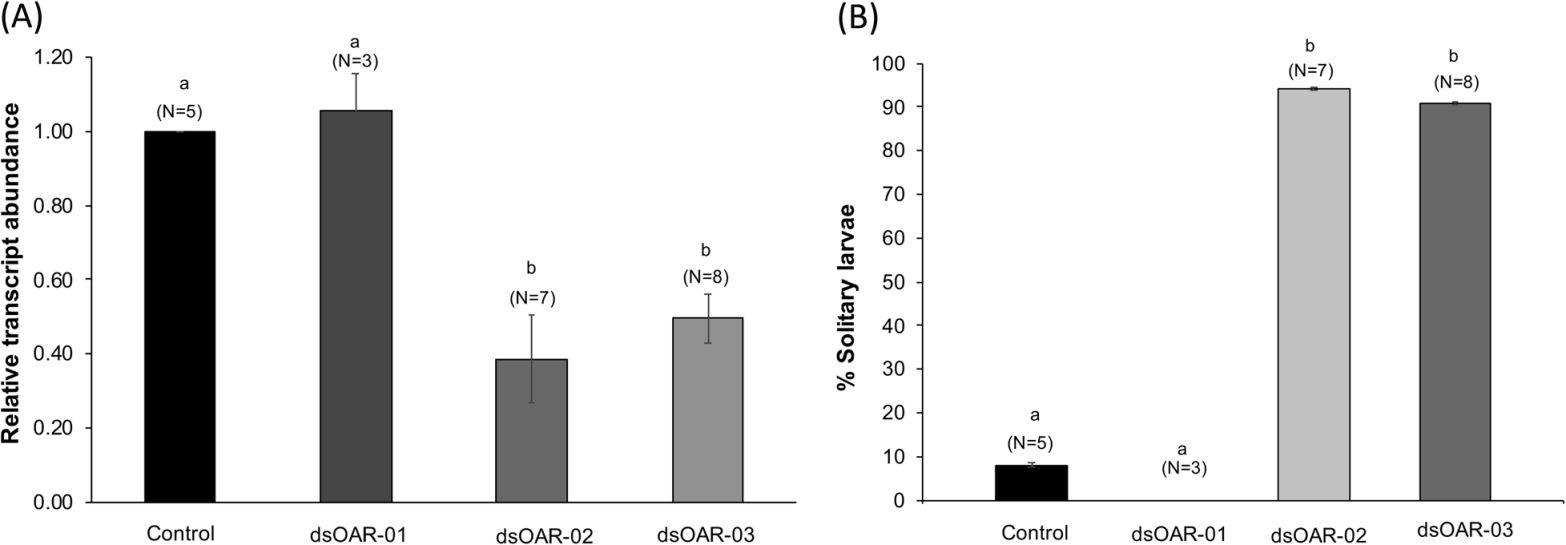
A decrease in DaOAR transcript abundance correlates to grouping behavior. Bars represent means ± S.E., bars with different letters are significantly different (Wilcoxon–Mann–Whitney). N = number of replicate trials. **A** Relative to the DEPC water control, a significant decrease in the DaOAR transcript abundance of octopamine receptor gene was observed at 48 h for dsDaOAR2 (p = 0.001) and dsDaOAR3 (p = 0.0004), but not with dsDaOAR1 (p = 0.642). Transcript abundance was quantified using RT–qPCR. **B** Percentage of larvae in control and dsRNA treatments that remained solitary at the end of behavioral trials. No significant differences were observed in control vs dsDaOAR-1 where none of the larvae remained solitary (p = 0.05). Significant differences observed in control vs dsDaOAR-2 (p = 0.001) and control vs dsDaOAR-3 (p = 0.0005)

Three dsRNAs (dsDaOAR-1, dsDaOAR-2, dsDaOAR-3) that target different regions of octopamine receptor transcript were separately fed to *D. arcuata* larvae to examine for evidence of RNAi in comparison to DEPC-water control diets (Additional file 1: Table S3). Leaf area consumed by each larva in DEPC-water controls and dsOAR-treatment trials was similar, 2.73 ± 0.15 mm^2^, equivalent to a dose of 0.082 µg (0.063–0.114 µg) of dsRNA per larva, assuming equal feeding by all larvae. Relative to DEPC-water control diets, RT–qPCR revealed a significant reduction of target octopamine receptor transcript at 48 h when larvae had been fed either dsDaOAR-2 or dsDaOAR-3, but no significant difference with dsDaOAR-1 feeding (Fig. 2). Octopamine receptor transcript abundance increased by 72 h in dsDaOAR-2 and dsDaOAR-3 treatments (Additional file 1: Table S1), indicating the transient nature of the dsRNA knockdown effects. Overall, the results indicate that 48 h of feeding larvae either dsDaOAR-2- or dsDaOAR-3-coated birch leaves results in a significant reduction of the target octopamine receptor transcript abundance (Fig. 2A).

#### Delayed behavioral transition correlates with reduced octopamine transcript abundance

To determine if caterpillars that were fed on dsDaOAR-1, dsDaOAR-2 and dsDaOAR-3 had different group-formation behavior compared to those fed on DEPC water control diets, observations were made during the 48–72 h period. Figure 2B shows that over 90% of the larvae fed with either dsDaOAR-2 or dsDaOAR-3 dsRNA remained solitary throughout this 24 h observation period, compared to less than 10% of those fed on dsDaOAR-1 or the water control. The solitary life-style exhibited by dsDaOAR-2- and dsDaOAR-3-treated 2nd-instar *D. arcuata* larvae is highly unusual in our extensive experience with *D. arcuata* [6]. Note also, that dsDaOAR-1 does not significantly induce RNAi (Fig. 2A) nor does it change timing of the behavioral shift (Fig. 2B). dsDaOAR-1 can be considered, then, as a negative control that shows that simply feeding dsRNA to the caterpillars does not alter the behavioral shift; an RNAi-induced decrease in octopamine receptor expression is apparently needed for the behavioral effect. At the end of group-formation trials, all treatments had equivalent numbers of caterpillars in 2nd instar (40%) or initiating a 2nd to 3rd instar molt, suggesting no differences in transition rates from 2nd to 3rd instar among treatments. These results support the hypothesis that downregulation of octopamine receptor gene in 2nd instars hastens the switch from social to solitary behavior.

### Discussion

Social behaviors in insects are complex and can be modulated by one or multiple genes and complex environmental cues [18]. The results presented here indicate that differential expression of octopamine receptor modulates the behavioral transition from group to solitary living in *D. arcuata* caterpillars. Octopamine has varied functions in insects, acting as a neuromodulator, neurohormone and a neurotransmitter [19], and previous studies have described the roles of octopamine receptors in mediating different behaviors, including social behavior [13, 14, 19–25]. Our current study extends the evidence that octopamine signaling acts as a driver of social behavioral transitions to the Lepidoptera. It should be noted that other biogenic amines such as dopamine and serotonin are also involved in mediating various insect social behaviours (e.g., [26–28]) and the interactions among the different amines including octopamine could be involved in shaping larval grouping behavior; however, exploring these molecular mechanisms is beyond the scope of this study. Our results confirm that the feeding dsRNA to Lepidoptera can trigger RNAi. Evidence for the efficacy of this method are few in Lepidoptera; however, it is not without precedence. For example, Gong et al. [29] fed dsRNA that targeted transcripts of the acetylcholinesterase gene to disrupt larval growth and development in the crop pest- diamondback moth, *Plutella xylostella,* providing an innovative strategy for developing RNAi-based pesticides. The findings of our current study broaden the potential applications of RNAi to unraveling the genetic mediation of social behaviors in Lepidoptera.

## Limitations

Sample sizes of caterpillars were limited for two reasons. First, caterpillars of this species must be reared from eggs obtained from wild-caught female moths. Both moths and host plants are seasonally limited. Second, the social/grouped phase of development is restricted to 3–5 days, reducing the availability of larvae of the same age.

## Supplementary Information

**Additional file 1: Table S1.** Positions and sequences of synthesized siRNAs targeting *D. arcuata* octopamine transcript sequence. **Table S2.** Sequences of RT–qPCR primers used in this study. **Table S3.** RT–qPCR and behavioral assay results for individual trials for dsRNA and water control.

## Data Availability

All the data generated and/or analyzed in this study are included in this manuscript and its additional files.
