# Supplementary material for: Octopamine receptor gene influences social grouping in the masked birch caterpillar
Source: BMC Res Notes. 2022 Jun 20;15:211. doi: 10.1186/s13104-022-06102-3 (PMC9208175; doi:10.1186/s13104-022-06102-3)
Supplement: Supplementary file 1 — Additional file 1: Table S1. Positions and sequences of synthesized siRNAs targeting D. arcuata octopamine transcript sequence. Table S2. Sequences of RT–qPCR primers used in this study. Table S3. RT–qPCR and behavioral assay results for individual trials for dsRNA and water control. [file 13104_2022_6102_MOESM1_ESM.docx]

Table S1. Positions and sequences of synthesized siRNAs targeting *D. arcuata* octopamine transcript sequence.

| **siRNA** | **Location relative to ATG start codon** | **Nucleotide sequence** |
| --- | --- | --- |
| dsOAR-1 |  |  |
| Sense | 83-103 | 5’- GUACCUCGUAUGCCCUCUAUA[dT][dT] -3’ |
| Anti-sense | 103-83 | 5’- UAUAGAGGGCAUACGAGGUAC[dT][dT] -3’ |
| dsOAR-2 |  |  |
| Sense | 740-760 | 5’- GGAUCCUAUCGCUUGUCAUUA[dT][dT] -3’ |
| Anti-sense | 760-740 | 5’- UAAUGACAAGCGAUAGGAUCC[dT][dT] -3’ |
| dsOAR-3 |  |  |
| Sense | 1467-1487 | 5’- GCUCCCAUUCUUCGUGAUUUA[dT][dT] -3’ |
| Anti-sense | 1487-1467 | 5’- UAAAUCACGAAGAAUGGGAGC[dT][dT] -3’ |

**Table S2.** Sequences of RT-qPCR primers used in this study.

| Primers | Nucleotide sequences |
| --- | --- |
| *DaOar* – F | 5’- TTCCGAGGTGTACGAGGTAA-3’ |
| *DaOar* – R | 5’- ACATATCGCTTCCCATTCTG-3’ |
| *DaRps7*– F | 5’- GTTGAAGGCATTCCAGAAGA-3’ |
| *DaRps7* – R | 5’- TAGGGAGAATCTTGCGGTCT-3’ |

Table S3. RT-qPCR and behavioral assay results for individual trials for dsRNA and water control.

|  | **Octopamine receptor transcript abundance relative to water control** | | | | **Behavioral assays during 48 – 72 h** | | | |  |
| --- | --- | --- | --- | --- | --- | --- | --- | --- | --- |
| siRNA/trial | 48 h | | 72 h | | Larvae in groups | | Solitary larvae | |  |
| dsOAR-1 |  | |  | |  | |  | |  |
| Trial 1 | 0.99 | | 0.98 | | 5 | | 0 | |  |
| Trial 2 | 1.25 | | 1.65 | | 5 | | 0 | |  |
| Trial 3 | 0.92 | | 2.27 | | 4 | | 0 | |  |
| MEAN ± SE | 1.05 ± 0.10 | | 1.63 ± 0.37 | | 4.67 ± 0.33 | | 0 | |  |
| p value^1^ | 0.643 | | 0.642 | |  | | 0.05 | |  |
| dsOAR-2 |  | |  | |  | |  | |  |
| Trial 1 | 0.12 | | 1.699 | | 0 | | 5 | |  |
| Trial 2 | 0.53 | | 0.710 | | 0 | | 5 | |  |
| Trial 3 | 0.08 | | 2.908 | | 0 | | 5 | |  |
| Trial 4 | 0.61 | | 0.444 | | 0 | | 5 | |  |
| Trial 5 | 0.89 | | 0.285 | | 2 | | 2 | |  |
| Trial 6 | 0.06 | | 0.392 | | 0 | | 5 | |  |
| Trial 7 | 0.41 | | 1.659 | | 0 | | 5 | |  |
| MEAN (± SE)  p value | 0.39 ± 0.18  0.001 | | 1.16 ± 0.56  0.682 | | 0.29 ± 0.44 | | 4.57 ± 0.65  0.001 | |  |
| dsOAR-3 |  | |  | |  | |  | |  |
| Trial 1 | 0.38 | | 0.785 | | 0 | | 5 | |  |
| Trial 2 | 0.22 | | 0.230 | | 0 | | 5 | |  |
| Trial 3 | 0.42 | | 0.225 | | 0 | | 6 | |  |
| Trial 4 | 0.50 | | 0.412 | | 0 | | 6 | |  |
| Trial 5 | 0.52 | | 0.613 | | 0 | | 5 | |  |
| Trial 6 | 0.72 | | 2.173 | | 2 | | 3 | |  |
| Trial 7 | 0.41 | | 1.266 | | 0 | | 6 | |  |
| Trial 8 | 0.80 | | 1.790 | | 2 | | 4 | |  |
| MEAN (± SE)  p value | 0.50 ± 0.11  <0.001 | | 0.94 ± 0.42  0.4 | | 0.50 ± 0.53 | | 5.00 ± 0.62  <0.001 | |  |
| DEPC Water Controls | |  | |  | |  | |  | |
| Trial 1 | NA | | NA | | 5 | | 0 | |  |
| Trial 2 | NA | | NA | | 5 | | 0 | |  |
| Trial 3 | NA | | NA | | 5 | | 0 | |  |
| Trial 4 | NA | | NA | | 5 | | 0 | |  |
| Trial 5 | NA | | NA | | 3 | | 2 | |  |
| MEAN (± SE) | NA | | NA | | 4.33 ± 0.65 | | 0.67 ± 0.67 | |  |

^1^p values in this table were calculated using Wilcoxon-Mann-Whitney test.

Values at 48 h and 72 h represent relative transcript abundance levels calculated using double delta Ct method. Values less than or greater than 1.0 indicate a reduction or increase, respectively, in octopamine receptor transcript abundance in treated vs control caterpillars. Group formation results are provided as the total number of larvae that formed groups or remained solitary. NA = not applicable.
